# Potential Analysis and Preparation of Chitosan Oligosaccharides as Oral Nutritional Supplements of Cancer Adjuvant Therapy

**DOI:** 10.3390/ijms20040920

**Published:** 2019-02-20

**Authors:** Zhiwen Jiang, Hui Li, Jing Qiao, Yan Yang, Yanting Wang, Wanshun Liu, Baoqin Han

**Affiliations:** 1 College of Marine Life Sciences, Ocean University of China, Qingdao 266100, China; jiangzhiwen@ouc.edu.cn (Z.J.); qiaojing@ouc.edu.cn (J.Q.); yany@ouc.edu.cn (Y.Y.); 15762182839@163.com (Y.W.); WanshunLiu@hotmail.com (W.L.); 2 Laboratory for Marine Drugs and Bioproducts of Pilot National Laboratory for Marine Science and Technology, Qingdao 266000, China; 3 Qingdao Biotemed Biomaterial Co., Ltd., Qingdao 266101, China; hblh2000@163.com

**Keywords:** chitosan oligosaccharide, anti-tumor, cytotoxicity, functional

## Abstract

Cancer is considered to have an adverse influence on health around the world. Chitosan, a linear polysaccharide that contains copolymers of β-1-4 linked d-glucosamine and *N*-acetyl-d-glucosamine units, has been widely used in the field of biomedicine, owing to its nontoxicity, biocompatibility, biodegradability, and hemocompatibility. This study was aimed at preparing the chitosan oligosaccharides (COS) and examining its ability on suppressing lung cancer in vitro and in vivo. Human non-small-cell lung cancer A549 cells model and C57BL/6 mice bearing lung cancer model were adopted. COS showed inhibition on the viability and proliferation of lung carcinoma cells (A549) in time-dependent manners, but no cytotoxicity to human liver cell (HL-7702). Moreover, COS could significantly increase Bax expression of A549 cells while decreasing Bcl-2 expression. COS supplementation significantly inhibited the growth of Lewis tissues and promoted necrosis of tumor cells in vivo. After treatment with COS, significantly elevated concentrations of Bax and reduced expression of Bcl-2 in tumor tissues, as well as elevated levels of TNF-α, IL-2, Fas and Fas-L in mice serum were observed (*p* < 0.05). In conclusion, COS had certain anti-tumor effects and potential application as a synergic functional food ingredient to prevent cancer.

## 1. Introduction

Cancer is one of the leading chronic diseases, with a high mortality rate worldwide [1]. Furthermore, problems associated with cancer are increasing in developing countries in line with population aging and the adoption of cancer-associated lifestyles [2]. Lung cancer is still the most common cause of cancer-related death [3]. The standard treatment method, a combination of surgery, radiotherapy, and chemotherapy, can kill cancer cells effectively, but is toxic to normal cells, which often impairs patients’ quality of life and is limited in tumor treatment [4]. In the past several decades, a number of new therapeutic strategies have been developed, including targeted therapy, hormonal therapy, and immunotherapy, all of which have been effective in treating certain types of cancer. However, long-term survival rates are still not satisfactory [5]. Natural products represent a rich source for the discovery and development of cancer-preventive and anticancer drugs. Chemoprevention by consumption of natural products of dietary origin has been employed as an important approach in the fight against cancer and has yielded promising outcomes [6]. Increasing demands of natural functional foods for health care and disease risk reduction are prevalent throughout the world [7].

Chitin, the most abundant natural polymer, except cellulose, in earth, is obtained from the shells of crab and shrimp [8]. Chitosan, a linear cationic polysaccharide that contains copolymers of β-1-4 linked d-glucosamine (GlcN) and *N*-acetyl-d-glucosamine (GlcNA) units, is obtained from the deacetylation of chitin and has been widely used in the field of biomedicine because of its nontoxicity, biocompatibility, biodegradability, and hemocompatibility [9]. Chitosan, due to the d-glucosamine, is insoluble at neutral and alkaline pH in aqueous solution but is soluble in dilute acids, such as acetic acid, formic acid, succinic acid, lactic acid, and malic acid, along with dilute hydrochloric acid [10]. Chitosan oligosaccharides (COS), with degrees of polymerization less than 20, are the depolymerized product of chitosan [11]. Due to the excellent water solubility, low molecular weights, low viscosity, and short chain lengths, COS is more suitable for some industrial applications and has gained increasing interest [12,13]. COS is a functional food with various biological activities, such as immune-stimulatory [14], antioxidant [15], anti-inflammatory [16], anti-carcinogenic [17], hypoglycemic [18], antimicrobial [19], and hypocholesterolemic [20] effects.

Previous experiments in vitro have revealed that COS induced the death of several cancer cell types, including ascites, leukemia, colorectal cancer, lung cancer, prostate cancer, bladder cancer, liver cancer, and cervical cancer [21,22,23,24,25,26,27]. COS had also been demonstrated to exhibit chemopreventive effects in rodent models of colorectal cancer and bladder cancer [28,29,30,31]. The results obtained by Kim et al. showed the correlationship of the degree of deacetylation (DD), molecular weight (MW), and anticancer activity on COS. The lower molecular weight COS has, the higher biological activity and higher degree of COS, which contained more amino group (NH_2_) and could show higher anticancer activity [32]. In the research, the highly efficient chitosanase prepared in our laboratory was used for hydrolysis of chitosan [33,34]. The objectives of this study were to provide a simple and efficient protocol for the preparation of COS with lower MW and investigate the anti-tumor action of COS on lung cancer in vitro and in vivo. Our data would provide more bases for developing effective and non-toxic dietary supplements against lung cancer.

## 2. Results and Discussion

### 2.1. Structures Characterization of COS

**Figure 1:**
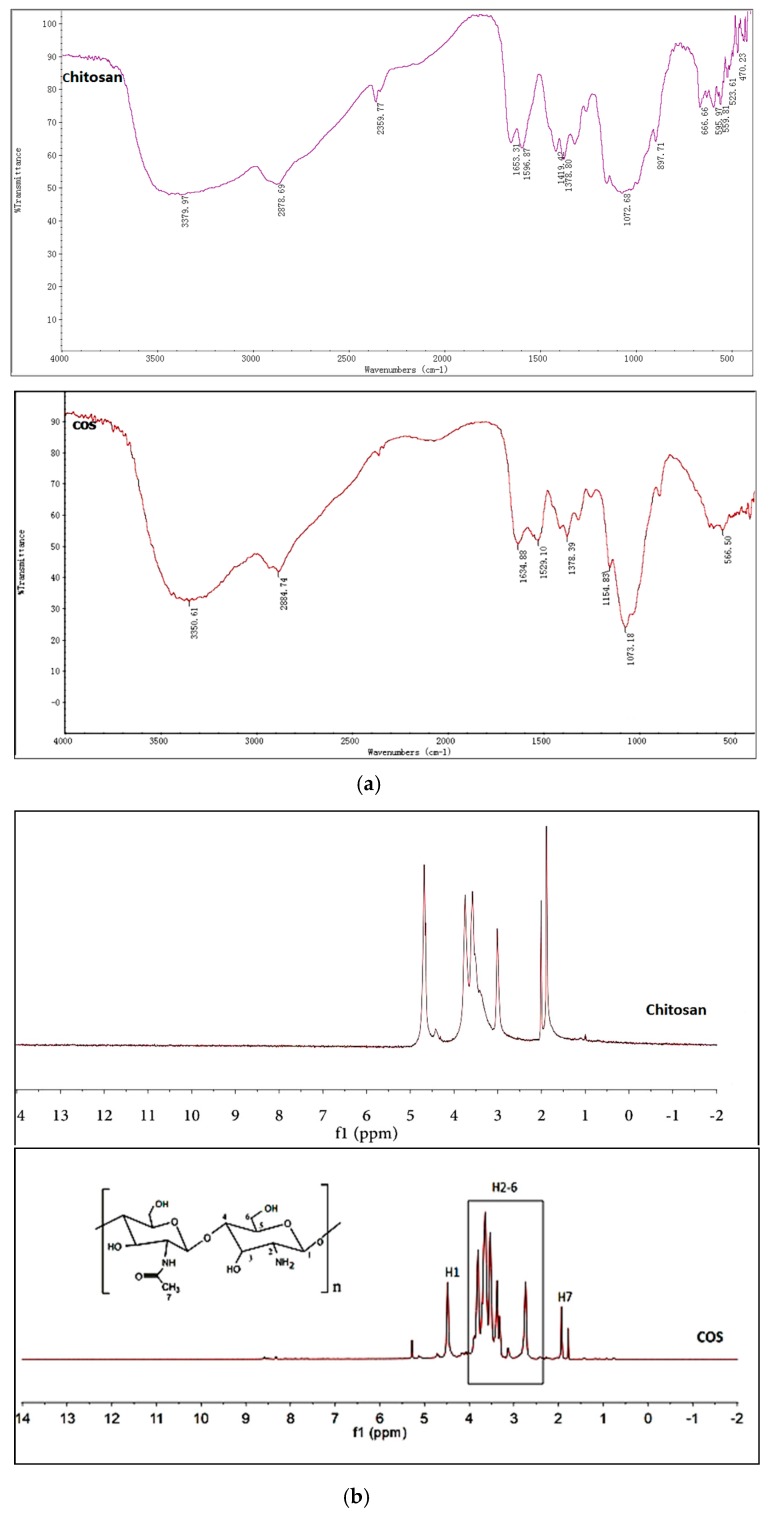

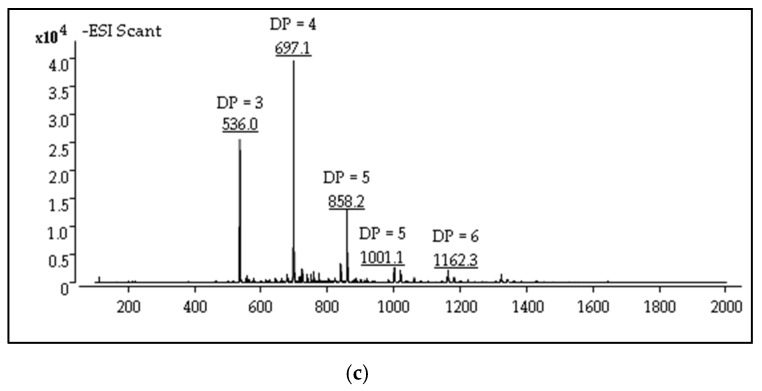
Infrared spectroscopy (IR) spectra (**a**), ^1^H nuclear magnetic resonance (NMR) spectra (**b**) and mass spectrum (MS) (**c**).

Enzymatic preparation methods for COS, most comparatively effective when compared to different traditional methods, have received great interest due to their safety and ease of control for enzymatic methods [35,36]. Based on previous studies of our laboratory, COS was obtained as a light-yellow powder with good water solubility at near-neutral pH. The chemical structures of COS were confirmed by fourier-transformed infrared spectroscopy (FTIR). As shown in Figure 1a, strong broad absorption peak at 3350 cm^−1^ was an overlap of N–H and O–H stretching vibration absorption peaks, which were sensitive to intermolecular hydrogen bonds. Peak at 2884 cm^−1^ was the absorption of C–H stretching vibration of methyl or methine. The FTIR spectrum of COS displayed peaks at 1634, 1529, and 1378 cm^−1^, corresponding to characteristic absorption peaks of amide I, amide II, and amide III bands. Furthermore, the absorption peak at 1073 cm^−1^ was the stretching vibration of C–O in the C-6 position. The ^1^H nuclear magnetic resonance (NMR) spectra of COS and chitosan was shown in Figure 1b comparison. The signals in the range of 2.0–5.0 ppm were assigned to the protons from amido linkage and pyranose ring of chitosan. The peaks between 2.5 and 4.1 ppm were ascribed to the protons of the glucosamine unit and the peak at 2.0 ppm was assigned to the methyl protons of the *N*-acetyl group in the ^1^H NMR spectrum of COS [37]. Compared with the results of chitosan used as raw material, the FTIR and ^1^H NMR spectra of COS showed no obvious peak differences. The characteristic groups of chitosan did not change during enzymatic hydrolysis. A decrease in the peak height corresponding to the protons (H7) of *N*-acetyl glucosamine (GlcNAc) (2.0 ppm) of COS, in comparison with those of native chitosan, further confirmed increased DD of the products. Oligosaccharides with different degrees of polymerization (DP) were identified in mass spectrum (MS) of COS (Figure 1c). The oligosaccharides with a DP of 3–6 comprised the main ingredients of the COS prepared from highly active chitosanase hydrolyzed chitosan. COS was identified with a water content of 7.51%, an ash content of 8.26%, and a purity of over 95%. The degree of deacetylation (DD) of COS, quantified with the acid–base titration method, was 93.33%. The heavy metal content and intracellular toxin content of COS were lower than 10 μg/g and 0.5 EU, respectively.

### 2.2. Different Effects of COS on the Viability of A549 and HL-7702 Cells

**Figure 2:**
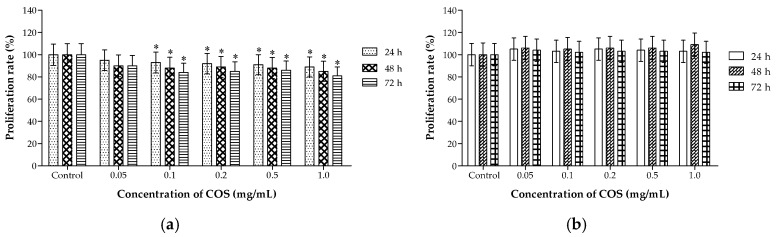
Effects of chitosan oligosaccharides (COS) on the proliferation of A549 and HL-7702 cells. (**a**) Proliferation rate of A549 cells measured by 3-(4,5-dimethylthiazol-2-yl)-2,5-diphenyl tetrazolium bromide (MTT) test. (**b**) Proliferation rate of HL-7702 cells measured by MTT test. Data represents mean ± SD, *n* = 6, * *p* < 0.05 significant difference compared with control group.

Studies have shown that a drug that can selectively cause damage to tumor cells with no toxicity on normal diploid cells is considered to be an ideal anti-cancer agent. COS is promising as a drug candidate and food ingredient, since they are naturally biocompatible, non-toxic, and non-allergenic to living tissues [38]. After incubation with COS for 48 h, human non-small-cell lung cancer A549 cells showed an unusual arrangement with reducing cell attachment numbers compared with the control group. After being incubated with various concentrations of COS for 48 h, human normal hepatocytes HL-7702 cells in each group had spread well and were full of cytoplasm with no obvious change. 3-(4,5-dimethylthiazol-2-yl)-2,5-diphenyl tetrazolium bromide (MTT) assay showed that COS had an obvious cytotoxic effect on the lung cancer A549 cells in time-dependent manners (Figure 2a). Cytotoxicity test was also assessed in HL-7702 cells after treatment for 24, 48, and 72 h (Figure 2b). COS did not show obvious inhibitory effects on cell viability. There was no statistical difference between treated groups and the control group. We evaluated the toxicity of COS by incubating cultured cancer cells and normal cells with different concentrations to obtain information about the potential chronic toxicity. COS could suppress human lung tumor growth without cytotoxicity on normal cells at the concentrations range of 0.05 to 1.0 mg/mL. In this study, COS was prepared as an oral nutritional supplement for lung cancer, and the water solubility, biosafety, and effectiveness in vivo are very important. Based on previous studies, COS was obtained as a light-yellow powder with good water solubility. As a sensitive normal cell line, human liver cell HL-7702 was used in vitro to detect the cytotoxicity of COS. COS had obvious cytotoxic effects on the lung cancer A549 cells, while COS did not show obvious inhibitory effects on the cell viability of HL-7702 cells. This thesis was focused on the preparation of nontoxic and effective agents. The different effects of COS on the viability of A549 and HL-7702 cells were highlights of the paper. COS is safe and effective for preventing cancer, which provided more data for the utilization of COS on oral nutritional supplements for lung cancer.

### 2.3. Growth Inhibiting and Apoptosis Promoting of COS on A549 Cells

**Figure 3:**
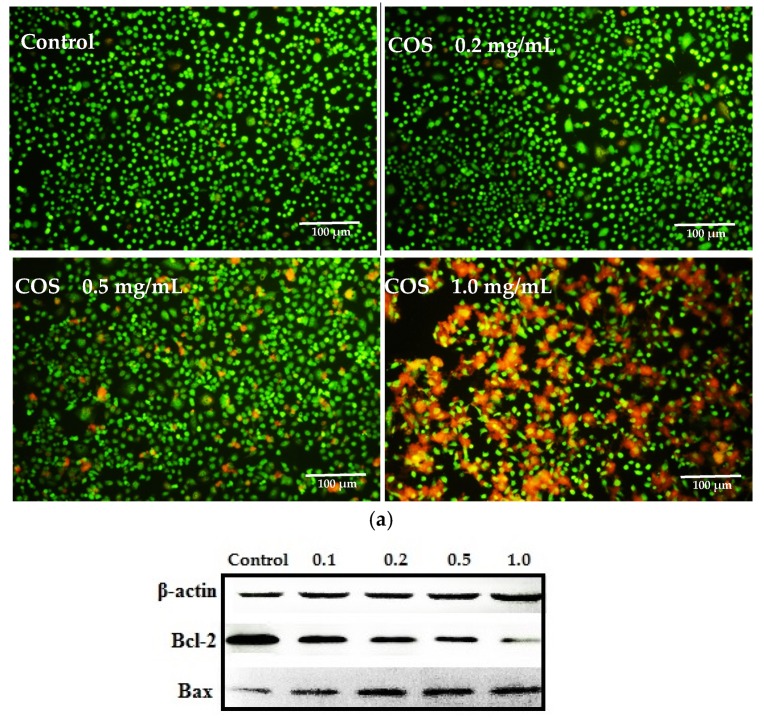

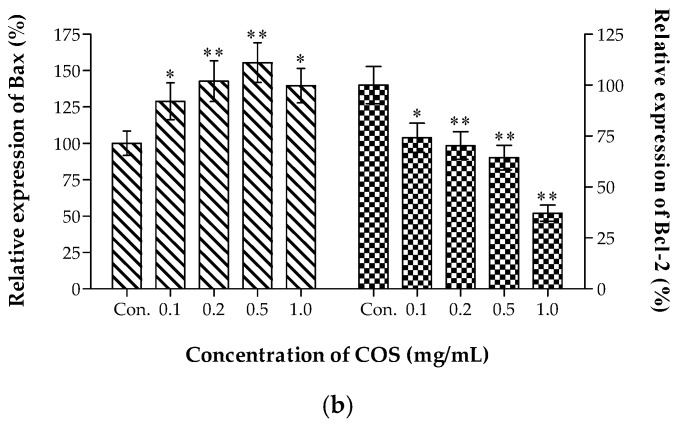
Effects of COS on cell viability and apoptosis of A549. (**a**) Morphological changes of A549 cells with Calcein-AM/PI assay (original magnification, 100×); (**b**) Relative expression of Bax and Bcl-2 with western blotting. Data represents mean ± SD, *n* = 6, ** *p* < 0.01 significant difference versus control group.

The inhibition of tumor growth has been a continuous effort in cancer treatment [39]. Growth reduction and death induction are two major means to inhibit tumor growth [40]. The effects of COS on cell viability were evaluated with live dead staining assay [41] on human lung cancer A549 cells. Healthy cells were stained by the cell-permeable Calcein-AM and fluoresced green, while dead cells were stained by the cell non-permeable PI (red) and appeared red. Low-dose COS (less than 0.2 mg/mL for 24 h) had slight toxic effects on the viability of A549 cells, while it was worthy to note that cell viability was highly affected by high-dose COS (0.5–1.0 mg/mL for 24 h) (Figure 3a). Members of the B cell lymphoma 2 (Bcl-2) gene family have a central role in regulating programmed cell death by controlling pro-apoptotic and anti-apoptotic intracellular signals. In cancer, apoptosis evasion through dysregulation of Bcl-2 genes is a recurring event [42]. As a pro-apoptotic member of this family of proteins, Bax is a critical regulator of apoptosis [43]. Therefore, we measured protein expressions of Bcl-2 and Bax in A549 cells using western blotting. As shown in Figure 3b, Bax expressed at a low level in the control group but was strongly activated by COS (*p* < 0.01), whereas the level of Bcl-2 expression was lower in COS-treated groups than that of control groups. Cell death, due to necrosis and apoptosis, was involved in COS treatment. These findings provided evidence that COS could cause significant growth inhibition in A549 human lung cancer cell lines and showed good ability at inducing cell apoptosis.

### 2.4. Effects of COS on Body Weight and Tumor Growth of Mouse

**Figure 4:**
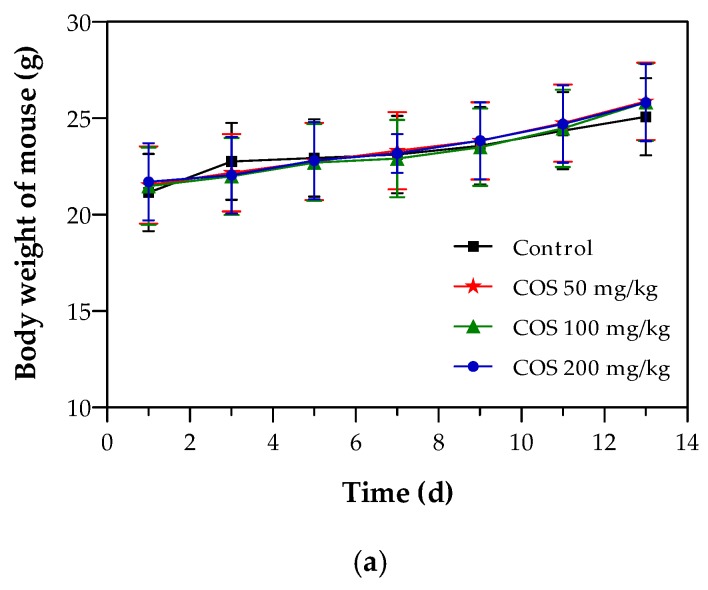

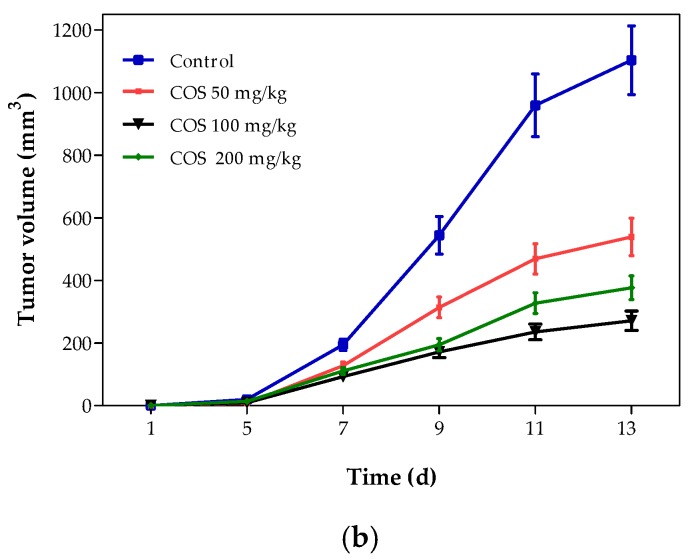
Effects of COS foods on the trend changes of body weight (**a**) and tumor volume (**b**) of tumor-bearing mice. Data represents mean ± SD, *n* = 12.

Oral route of administration is the most convenient and common and is preferred for clinical therapy. Many drugs have been prepared in oral dosage form. Moreover, COS was prepared as a functional food or dietary supplement to prevent cancer, so the mice were orally administrated in this study. The body weight of each mouse was weighed every other day (Figure 4a). No significant differences were observed in the body weights among the different groups throughout the experimental period (*p* > 0.05). While all the mice weighed roughly 20 percent more from the first day to the last day, there was no distinct difference in the body weight changes after COS treatment compared with the control group, intimated that there was no drug-induced toxicity in the dosage instructions given [44]. Therefore, the inhibition of COS on Lewis solid tumor did not rely on toxicity. To assess the effect of COS on lung tumor growth, mean tumor volumes were calculated and compared between the control group and different treated groups (Figure 4b). As early as 7 days after inoculation, COS inhibited tumor cell growth had a conspicuously small tumor volume compared to the control (*p* < 0.05). 13 days later, the treated groups, especially at intermediate and high doses, showed significant reductions in the tumor volume, which was different to the control group. Overall, the anti-proliferative effect of COS on the growth of lung tumor in mice was achieved in a time-dependent manner.

Lewis tumor-bearing mice, a suitable in vivo model, can be used for the evaluation of anti-cancer drug efficacy and the exploration of mechanisms of drug metabolism in human lung cancers. Lewis cells isolated from C57BL/6 mice were initially highly metastatic tumors cells. Upon the subcutaneous graft, the cells developed into tissue in a semi-firm homogeneous mass [45]. According to the results of Janker et al., the tumor incidence rate with Lewis subcutaneous injection reached 100% without mortality [46]. To assess the effects of COS on lung tumor growth, the weight of the tumor tissue was measured after experiment. As shown in Table 1, significant reductions of the tumor weights were observed in COS-treated mice compared with the control group (*p* < 0.05). Results displayed that inhibition rates of the Lewis solid tumor were 35.32%, 52.53%, and 48.44% at doses of 50, 100, and 200 mg/kg, respectively. Furthermore, COS at intermediate and high doses appeared to be more effective for restricting tumor growth when compared with the low dose group. Therefore, the in vitro and in vivo results revealed that COS possessed potent anti-cancer activity and exerted the anti-tumor effects without toxic influences on normal cells or body weight.

### 2.5. Effects of COS on Histopathological Changes of Tumor and Lung Tissue

**Figure 5:**
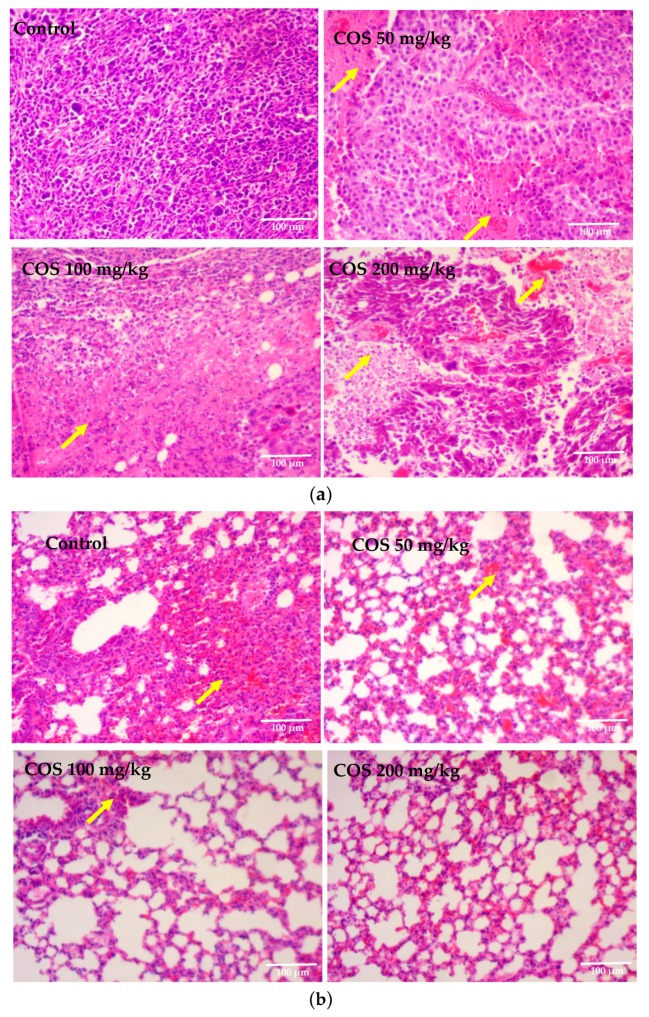
Effect of COS on the histopathology of Lewis tumor and lung tissues (original magnification, 100×). (**a**) Pathological section of tumor; (**b**) Pathological section of lung tissues. Arrow: apoptosis cells.

Tumor tissues of Lewis tumor-bearing mice were processed for H-E staining to determine the presence of apoptosis cells. In COS treatment groups, a relatively lower nuclear to cytoplasmic ratio in the tumors was observed compared to the control group. Marked changes in the morphology of the nucleus in COS-treated groups were also observed, such as karyorrhexis and nucleus fragmentation. Conversely, the individual cells in the control group were clearly visible in appearance, in which no obvious morphological changes were observed (Figure 5a). The morphological changes of lung tissues treated with COS were evaluated by H-E staining. As shown in Figure 5b, lung cells in the control group arranged in disorder, obvious edemas, and vacuolar degeneration could be seen. While in COS treatment groups, lung tissues exhibited relieving in the edema formation and vacuolar degeneration. H-E staining demonstrated that COS administration increased apoptosis of the tumor and repaired lung injuries in Lewis tumor-bearing mice.

### 2.6. Effects of COS on the Expression of Bax and Bcl-2 In Vivo

**Figure 6:**
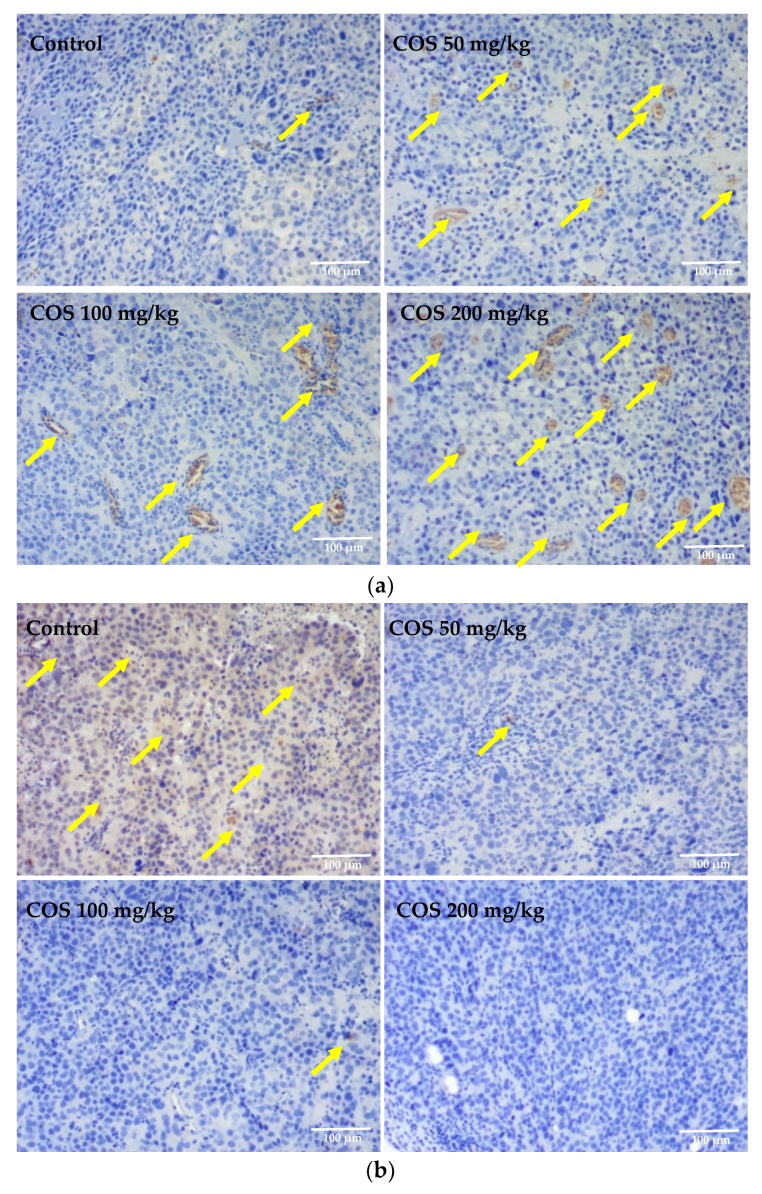

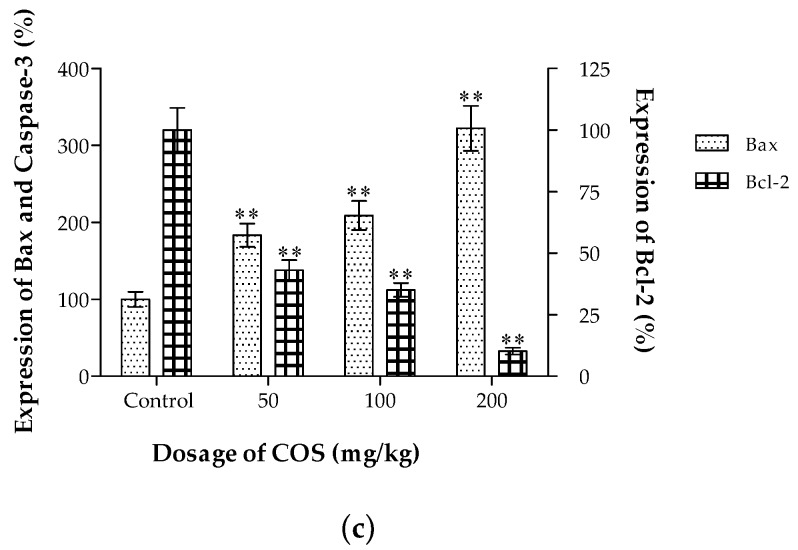
Immunohistochemical detection on Lewis tumor tissue (original magnification, 100×). (**a**) Photographs of Bax expression in Lewis tumor tissue; (**b**) Photographs of Bcl-2 expression in Lewis tumor tissue; (**c**) Effects on Bax and Bcl-2 expression of Lewis tumor tissue were quantified by Image Pro Plus software. The relative average optical density of the control group was adjusted to 100%. Data represents mean ± SD, *n* = 12, ** *p* < 0.01 significant difference compared with control group. Arrow: high expression of immune proteins.

Apoptosis plays a crucial role in the suicide and turnover of cells in various tumors [47]. The chemical-induced apoptotic pathway involved in mitochondria has been shown to be regulated by key proteins, such as Bax and Bcl-2 [48]. Bcl-2, predominantly localizing on the outer mitochondrial membrane, contains a hydrophobic cleft, which can bind to the propoptotic Bcl-2 family members, such as Bax, to inhibit apoptosis [49]. The Bcl-2 protein mediates anti-apoptotic effect by stabilizing the mitochondrial membrane and inhibiting permeability transition pore ability [50]. In contrast, the Bax protein predominantly localizes in the cytosol, and, upon activation, translocates to the mitochondria and triggers the loss of mitochondria membrane potential [51]. The Bax protein serves as a positive regulator of apoptosis by forming heterodimers with Bcl-2 protein, thereby promoting cell survival [52]. To determine the effects of COS on the expression of apoptosis-associated proteins in vivo, the contents of Bax and Bcl-2 in the tumors were detected with immunohistochemical staining. The results, under optical lens positive products, were brown particles and strong positive staining, which might correspond to high expression of immune proteins. As shown in Figure 6a,b, compared with the control group, the expression levels of Bax increased in the COS-treated groups, while the levels of Bcl-2 expression decreased. As shown in Figure 6c, the expression of Bcl-2 was dramatically decreased in COS-treated groups in a dose-dependent manner, which showed significant differences with the control (*p* < 0.01). Meanwhile, the expressions of Bax in the COS-treated groups were significantly up regulated compared with the control group (*p* < 0.01). Collectively, we found that the expression of Bax increased and the expression of Bcl-2 reduced significantly following COS treatment, indicated that COS administration could induce apoptosis in Lewis tumor-bearing mice.

### 2.7. Effects of COS on TNF-α, IL-2, Fas and Fas-L Levels in Serum

**Figure 7:**
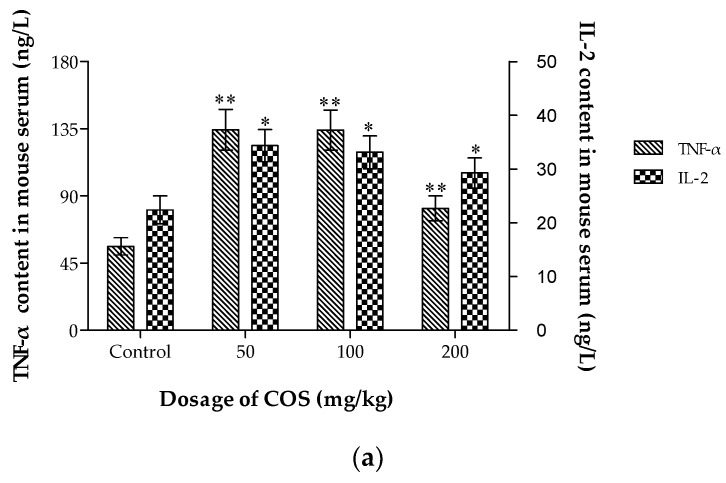

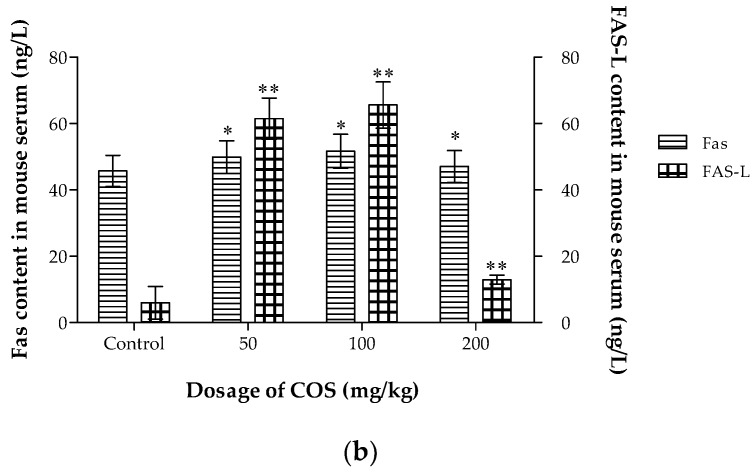
Effect of COS on serum TNF-α, IL-2 (**a**), and Fas, Fas-L (**b**) levels. Data represents mean ± SD, *n* = 12, * *p* < 0.05, ** *p* < 0.01 significant difference compared with control group.

Different types of lymphocytes have different roles in tumor suppression [53]. TNF-α acts as a key mediator for local inflammation and cancer development. TNF-α can enhance host immune function and induce the apoptosis of tumor cells [54]. IL-2 has been used extensively in murine cancer models and human clinical trials due to its effects in activating different effector components of anti-tumor immune responses [55]. The Fas-FasL system plays a crucial role in the counterattack of cancer cells against the immune system [56]. Fas belongs to the TNF/nerve growth factor receptor family, and it mediates apoptosis [57]. Fas-L, upon engaging and activating the Fas receptor on the surface of infected cells, delivers a death signal [58]. To assess cytokine secretions associated with the anti-tumor activities induced by COS in Lewis tumor-bearing mice, the cytokines levels in the serum were measured with ELISA kits (Figure 7). Treatment of the mice with COS (50, 100, 200 mg/kg) promoted TNF-α, IL-2, Fas, and Fas-L production in serum in Lewis tumor-bearing mice (*p* < 0.05). The results in the present study indicated that the immunomodulatory effects and the apoptosis-inducing function might be associated with the anti-tumor activity of COS. However, whether immunomodulation played an important role in COS-inhibited tumor growth was required to be investigated with more detail in the future.

In order to understand its internalization pathways, the pinocytosis of COS by macrophages was observed by fluorescence microscope. The results showed that FITC-COS could be phagocyted by macrophages. With the prolonged period, the fluorescence intensity enhanced. The results had been shown in Figure S1 of supplementary data. As complement of Figure 4, the original tumor size photos had been shown in Figure S2 of supplementary data.

## 3. Materials and Methods

### 3.1. Materials and Reagents

Chitosan (DD > 90%) was purchased from Qingdao Biotemed Biomaterial Co., Ltd. (Qingdao, China). Chitosanase was prepared, purified, and identified in our laboratory. Fetal bovine serum (FBS) and RPMI 1640 medium were obtained from Gibco^®^, Life Technologies (Carlsbad, CA, USA). Calcein-AM was purchased from Fanbo Biochemical Co., Ltd. (Beijing, China). The 3-(4,5-dimethylthiazol-2-yl)-2,5-diphenyl tetrazolium bromide (MTT) and propidium iodide (PI) were obtained from Sigma–Aldrich (St. Louis, MO, USA). The RIPA lysis buffer and the sodium dodecyl sulfate polyacrylamide gel electrophoresis (SDS-PAGE) gel preparation kit were obtained from Beyotime Biotechnology Co., Ltd. (Shanghai, China). The 3,3-diaminobenzidine (DAB) reagent kit, mouse tumor necrosis factor α (TNF-α) enzyme-linked immune sorbent assay (ELISA) kit, Mouse interleukin 2 (IL-2) ELISA Kit, Mouse Fas ELISA Kit and the Mouse Fas-L ELISA Kit were purchased from Boster Biological Engineering Co., Ltd. (Wuhan China). The rabbit polyclonal anti-Bcl-2 antibody, rabbit polyclonal anti-Bax antibody and goat anti-rabbit IgG Horse Radish Peroxidase (HRP) were purchased from Abcam Inc (Cambridge, MA, USA). Polyvinylidene difluoride (PVDF) membranes and bovine serum albumin (BSA) were obtained from Solarbio Science and Technology Co., Ltd. (Beijing, China).

### 3.2. Cell Lines and Animals

Human non-small-cell lung cancer (NSCLC) A549 cells, HL-7702 cells, and mouse lung cancer cell line Lewis were obtained from the Institute of Pharmacology, Ocean University of China. Cells were cultured in 1640 medium, supplemented with 10% FBS, 100 unit/mL penicillin, and 100 μg/mL streptomycin at 37 °C under a humidified atmosphere (5% CO_2_ plus 95% air). When cells reached 70–80% confluence, they were trypsinized, centrifuged, counted and seeded in cell plates. Eight-week-old C57BL/6 mice (half males and half females, 18–22 g), with the animal license of SCXK (Xiang) 2016-0004, were offered by the Hunan SJA Laboratory Animal Co., Ltd., (Changsha, China), and maintained in the animal care facility of the Biochemistry Laboratory of Ocean University of China. All animals were kept under a 12 h light–dark cycle at a consistent temperature (25 ± 3 °C) and relative humidity (60–70%). Experiments were performed in accordance with the ethical guidelines of the Shandong Province Experimental Animal Management Committee and were in complete compliance with the National Institutes of Health Guide for the Care and Use of Laboratory Animals (9787030482617, 1 May 2016).

### 3.3. Synthesis and Characterization of Chitosan Oligosaccharide

COS were prepared and purified as previously reported in our laboratory [59]. Chitosan was dispersed in distilled water and pH was regulated to 5.5. To make COS, the chitosanase was added to the chitosan solution in the ratio of 10 U/g. The mixture was stirred magnetically at 45 °C for 3 h and then the inactive chitosanase was removed using the method of sedimentation by trichloroacetic acid. After the reaction, the pH value of hydrolysate was adjusted to neutral with sodium hydroxide solution. COS was obtained by spray drying, desalted using membrane filtration, and purified with ethanol. The structure of COS was determined by FTIR, MS, and ^1^H NMR. The FTIR spectrum of chitosan and COS (~0.5 mg) was acquired on the NEXUE 470 instrument (Nicolet Co., Madison, WI, USA) as KBr pellets at room temperature. The MS of COS was detected by the Agilent 6460 Triple Quad LC/MS system (Agilent technologies Inc., Santa Clara, CA, USA). The ^1^H NMR spectra of chitosan and COS were recorded on a DPX300 spectrometer (Bruker, Rheinstetten, Germany) using acetone as an internal standard and 1% (*v*/*v*) DCl D_2_O solution and D_2_O as solvent, respectively, at 25 °C. The acid–base titration method was used to determine the deacetylation degree of COS.

### 3.4. Cell Proliferation and Viability Assays In Vitro

A549 or HL-7702 cells were seeded in 96-well flat-bottom culture plates at a density of 5 × 10^3^ cells/well and were allowed to grow with adherence overnight. Different concentrations of COS (0.05–1.0 mg/mL) were added and incubated for 48 and 72 h. The morphology and vitality of both cell lines were obtained from photographs under inverted microscope (T1-SM 100, Nikon Co., Tokyo, Japan) and MTT assay at each time point after treatment. MTT (5 mg/mL, 20 μL) was added to each well of 96-well flat-bottom culture plates. After 4 h of incubation with MTT, the medium was removed and 150 μl of dimethylsulfoxide was added. The plate was then read at 492 nm on the Multiskan Go 151 Microplate Scanning Spectrophotometer (Thermo Fisher Scientific, Inc., Waltham, MA, USA). Changes of cell activity were calculated using the percentage of proliferation rate (PR/%) according to the literature [60]. Each assay was repeated three times and all experiments were performed in sextuplicate wells.

### 3.5. Live-Dead Cell Staining Assay and Apoptosis Analysis

Calcein-AM/PI, the live/dead cell marker, was used according to the manufacturer’s protocol. A549 cells (2 × 10^5^ cells /mL) were exposed to COS (0.05–1.0 mg/mL) for 24 h under standard conditions, washed twice with phosphate buffered saline (PBS), and stained with 2.0 μmol/L Calcein-AM and 4.0 μmol/L PI for 30 min in the dark. Cells incubated with culture medium were used as the control group. Finally, all cells were washed twice using PBS and immediately observed under a fluorescence microscope using a band–pass filter.

The protein expression of Bcl-2 and Bax was detected by western blotting [61]. The A549 cells treated with COS (0.1, 0.2, 0.5 and 1.0 mg/mL) for 48 h were homogenized in 0.2 ml RIPA lysis buffer containing protease inhibitors, and were centrifuged at 10,000× *g* for 20 min at 4 °C. Then, the protein concentrations in the supernatants were determined with BCA method and adjusted to the same level using PBS and 4× loading buffer. The samples containing 40 μg of total protein were boiled for 10 min at 98 °C and subjected to electrophoresis on a 12% SDS-PAGE gel. Proteins on the gel were transferred to PVDF membranes, which were blocked with 5% BSA in PBS at room temperature for 1 h. The membranes were then incubated at 4 °C overnight with a 400 dilution of the Bcl-2 or Bax antibody, followed by incubation with a secondary HRP-conjugated antibody (1:5000) for 2 h at room temperature. Bands were visualized using DAB. Cells incubated with complete medium only were used as control. Bax protein expression was quantified by Image J software and the relative gray value of control group was adjusted to 100%.

### 3.6. Anti-Tumor Activities of COS in Lewis Tumor-Bearing Mice

The Lewis lung tumor model was established by the subcutaneous injection of Lewis cells (3 × 10^6^ cells/200 μL normal saline) into the right flank of each C57BL/6 mouse. Twenty-four hours after inoculation, the animals were weighed and randomly divided into four groups (*n* = 12). Mice in the control group were orally administrated with the menstruum saline. COS in normal saline (50, 100, 200 mg/kg) was infused to the stomachs of experimental mice every day for fourteen times. The body weight and tumor size of each mouse in all four groups were measured simultaneously. The equation tumour volume = (length × width^2^)/2, was used to calculate tumour size. Twenty-four hours after the last administration, the blood of each animal was collected through retro–orbital puncture. All animals were sacrificed and tumour tissues were excised and weighed to calculate anti-sarcoma rate. The inhibition of COS on the growth of Lewis tumor was calculated using the percentage of inhibition rate (IR). The IR was calculated according to the formula below: IR (%) = [1 − (Mean weight value of COS-treated animals/Mean weight value of control animals)] × 100%.

### 3.7. Histological and Immunohistochemical Analysis

Tumor and lung tissues from each group were fixed in 4% paraformaldehyde solution for 24 h, and embedded in paraffin for sectioning. For each sample, the histopathological alterations of consecutive 4 μm-thick sections were observed by Hematoxylin-Eosin (H-E) staining to examine the pathological changes with an optical microscope (E200, Nikon Co., Tokyo, Japan). For each sample of tumor tissues, the slides had immunohistochemical staining [62] with antibodies specific for the apoptotic cells. Sections were incubated with 3% hydrogen peroxide for 10 min to quench endogenous peroxidase, and antigen retrieval was completed by boiling in sodium citrate buffer (pH 6.0) for 10 min. Nonspecific protein-binding sites were blocked by 20-min incubation with 5% BSA in PBS. The slices were then incubated with the primary anti-Bcl-2 (1:200 dilution) or anti-Bax (1:150 dilution) at 4 °C overnight. After a further three rinses with PBS, sections were incubated with HRP conjugated goat anti-rabbit IgG for 30 min at 37 °C. The color was allowed to develop by incubating the samples for 5–10 min with chromogen DAB. The sections were counterstained with hematoxylin. Slides were observed and photographed using the E200 microscope (Nikon Co., Tokyo, Japan) and the DT300 digital camera software.

### 3.8. Measurement of TNF-α, IL-2, Fas and Fas-L Levels in Serum

Serum was collected from retrobulbar venous plexus of different groups by centrifugation (2000× *g*). Commercial ELISA kits were used for detection of TNF-α, IL-2, Fas and Fas-L according to the instruction manual. 100 μL of serum and standards were added to a 96-well Elisa plate coated in advance, and the mixtures were inoculated for 90 min at 37 °C. After washing with PBS, biotin labeled anti-IgG antibody was added into the wells and inoculated for another 60 min at 37 °C. Then after washing with PBS, the levels of cytokines in serum of mice were detected with HRP-streptavidin. The absorbance was read at 450 nm with a reference of 630 nm using a Multiskan Go 151 Microplate Scanning Spectrophotometer. The cytokine levels were calculated from standard curves of recombinant cytokines using the linear regression method [63].

### 3.9. Statistical Analysis

All data was expressed as mean ± SD. Data analysis was carried out by the Student’s *t* test or the one-way analysis of variance (ANOVA) with the use of *t* SPSS version 18 (SPSS Inc, Chicago, IL, USA). * *p* < 0.05 was set as significant level and ** *p* < 0.01 was considered as very significant.

## 4. Conclusions

Consumption of functional food or dietary supplements to prevent cancer has become more and more intriguing. COS, with good bio-safety to normal liver cells and mice with lung cancer, could directly inhibit tumor cell growth in time-dependent manners and induce apoptosis of A549 cells. Moreover, COS potentially inhibited the development of Lewis lung tumors in C57BL/6 mice. The effects of COS were likely to be achieved through the initiation of Bax and Bcl-2 mediated apoptosis. Furthermore, serum levels of TNF-α, IL-2, Fas and Fas-L were enhanced significantly when compared with the control group. The results showed that the enzymatic hydrolysates of chitosan played a significant role in limiting the growth of lung tumor in vitro and in vivo. These findings suggested that COS could be a promising alternative supplementation as adjuvant treatment in lung cancer.

## Figures and Tables

**Table 1:** Effects of COS on the Lewis tumor weight.

Group and Dosage	Weight of Tumor (g)	Inhibition on Tumor Weight
Control	1.71 ± 0.24	–
COS (50 mg/kg)	1.11 ± 0.37 *	35.32%
COS (100 mg/kg)	0.81 ± 0.18 **	52.53%
COS (200 mg/kg)	0.88 ± 0.21 **	48.44%

Data represents mean ± SD, *n* = 12, * *p* < 0.05, ** *p* < 0.01 significant difference compared with control group.

## Supplementary Materials

Supplementary materials can be found at https://www.mdpi.com/1422-0067/20/4/920/s1.
