# Supplementary material for: Potential Analysis and Preparation of Chitosan Oligosaccharides as Oral Nutritional Supplements of Cancer Adjuvant Therapy
Source: Int J Mol Sci. 2019 Feb 20;20(4):920. doi: 10.3390/ijms20040920 (PMC6412339; doi:10.3390/ijms20040920)
Supplement: Supplementary file 1 [file ijms-20-00920-s001.pdf]

The pinocytosis of COS by macrophages was observed by fluorescence microscope. The results showed that FITC-COS could be phagocyted by macrophages. With the prolonged period, the fluorescence intensity enhanced. The results were shown in **Figure S1**.

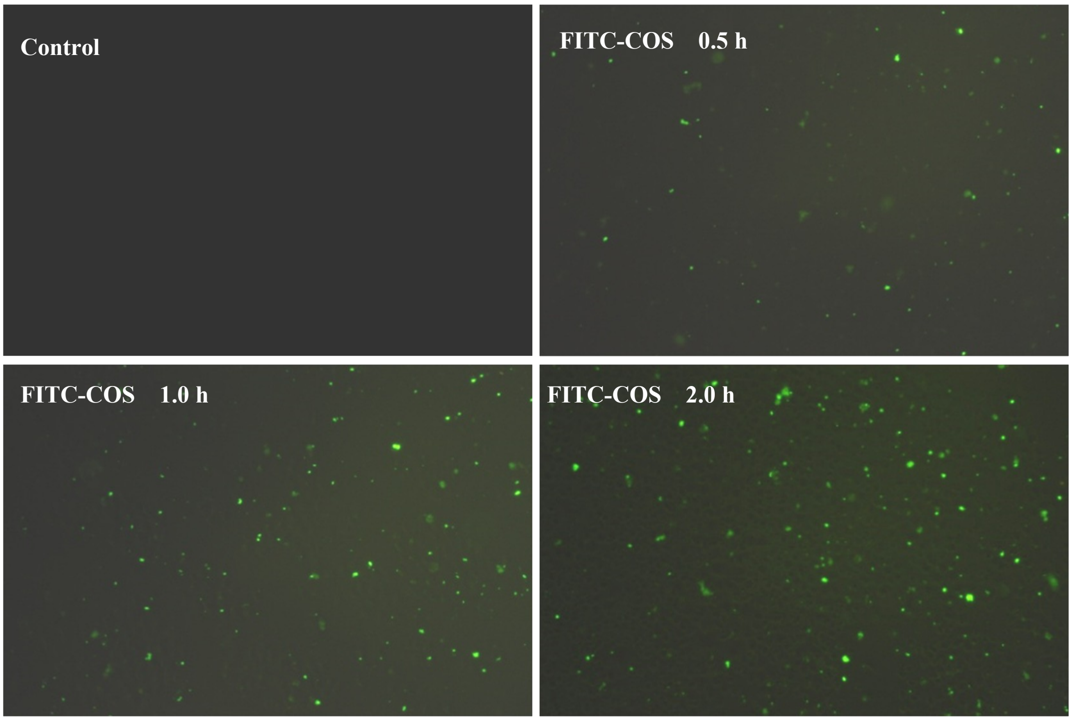

Figure S1. FITC-COS pinocytosis by macrophages. FITC-COS (0.5 mg/ml) in PBS was added to macrophages for different time of co-incubation. After co-incubation at different time intervals, cells were washed with PBS for three times and were observed with Nikon fluorescence microscope.

The original tumor size photos were shown in **Figure S2**.

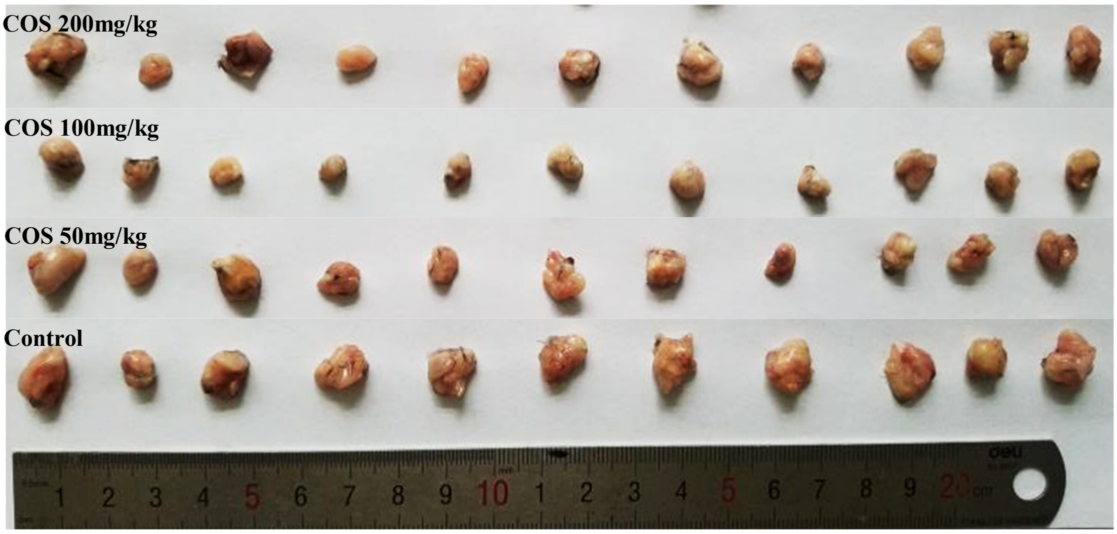

Figure S2. The photos of original tumor size.
